# Cranial Vault Suture Obliteration in Relation to Age: An Autopsy-Based Observational Study

**DOI:** 10.7759/cureus.39759

**Published:** 2023-05-31

**Authors:** Hitesh Chawla, Shiv Shankar, Ashish Tyagi, Jyoti Panchal

**Affiliations:** 1 Forensic Medicine, Shaheed Hasan Khan Mewati Government Medical College, Nalhar, Nuh, IND; 2 Forensic Medicine, Civil Hospital, Palwal, Palwal, IND; 3 General Practice, Primary Health Centre (PHC) Mohna, Faridabad, IND

**Keywords:** lambdoid, sagittal, coronal, endocranial, ectocranial, cranial sutures

## Abstract

Background: Age is one of the most critical identifiers for both living and dead. Forensic professionals in medical and legal matters are often presented with dismembered, disfigured, putrefied, or skeletal remains for analysis. In such situations, it is essential to identify individuals and estimate their ages. The skull is typically the well-preserved part of the body in such situations. If an aged person needs their age officially established for employment, superannuation, pension settlements, senior citizen benefits, etc., they may turn to medical professionals for help in making that determination. It has always been controversial to use cranial suture obliteration as a reference for age. Different geographical locations have been shown to have vastly different patterns of cranial suture closure. Therefore, this study was conceptualized to assess cranial vault suture obliteration in relation to age in the Meo population. This study was conducted to determine whether obliteration of cranial sutures can be taken into account for the estimation of age in elderly in this region and its reliability along with the influence of other factors such as sex and right and left side differences.

Materials and methods: A total of 100 cases of more than 20 years of age brought for medicolegal autopsy were analyzed. The coronal, sagittal, and lambdoid sutures were studied ectocranially and endocranially. The degree of obliteration of sutures was scored ectocranially as well as endocranially. Data were analyzed using IBM SPSS Statistics for Windows, Version 21 (Released 2012; IBM Corp., Armonk, New York, United States). Descriptive statistics were evaluated for continuous data in terms of mean and standard deviation, and categorical data were presented by frequency and percentages. An independent t-test was applied to find out the mean difference between the right and left sides of suture closure for ectocranial and endocranial surfaces. The Spearman rank correlation test was carried out to find out the relationship between the age and score of suture closure both ectocranially and endocranially.

Result: Ectocranially and endocranially, the overall sagittal suture obliterates early followed by coronal sutures and then lambdoid sutures. On comparing the mean ectocranial and mean endocranial scores of 100 subjects by applying an independent t-test, a highly significant difference was observed in all three sutures. On correlating ectocranial sutures and endocranial sutures and age at death in all the cases through sagittal, right and left coronal, and lambdoid by applying the Spearman rank correlation coefficient, a highly significant correlation was found in all the subjects combined (p-value 0.000). However, no significant correlation (p-value >0.05) was found in ectocranial and endocranial sagittal sutures in individual age groups.

Conclusion: We concluded that obliteration on the endocranial surface is more reliable than on the ectocranial surface. No statistically significant difference exists on the obliteration of sutures on the right and left sides of coronal and lambdoid sutures. The lapsed union was evident in all three sutures ectocranially. Endocranial suture obliteration can be used as a corroborative tool for age estimation.

## Introduction

Age is one of the most critical parameters for the identification of an individual, whether dead or alive. Determination of age depends on physical or morphological features, dental study, ossification activity, and growth of bones [1]. Based on the order of epiphysis-diaphyseal fusion of long bones and eruption and development of teeth, age estimation can be determined with certainty up to the age of 25 years. General physical characteristics like greying of body hairs, arcus senilis along with ossification of xiphoid and manubrium with the body of sternum, laryngeal and costal cartilages, hyoid bone, lipping of vertebrae, and obliteration of cranial sutures are the criteria which are taken to account for determination of age in middle and old age [1-4]. Many times, dismembered, mutilated bodies, putrefied bodies, and skeletal remains are produced before forensic experts for medico-legal examination. Other means of skeletal age estimation in old age such as hyoid bone, sternum, ossification of laryngeal, tracheal, and costal cartilages and general physical characteristics are not available in skeletal remains/mutilated bodies. The forensic expert then has limited resources to estimate the skeletal age of the deceased, which is then solely based on the available bones, and the skull is found to be the well-preserved remains in most cases. Medical opinion is also sought for the estimation of age during life in elderly people when fixing of age is required for regularization of employment, superannuation, pension settlements, senior citizen benefits, etc. Age estimation by cranial suture obliteration has always been a matter of debate. Much variation has been observed in the closure of cranial sutures in various geographical regions. Therefore, this study is conceptualized to assess cranial vault suture obliteration in relation to age in the Meo population. This study was conducted to determine whether obliteration of cranial sutures can be taken into account for the estimation of age in elderly in this region and its reliability along with the influence of other factors such as sex and right and left side differences.

## Materials and methods

The cross-sectional, observational study was carried out in the Department of Forensic Medicine at a tertiary care teaching institution (Shaheed Hasan Khan Mewati Government Medical College, Nalhar, Nuh) located in Southern Haryana. The approval of the Institutional Ethics Committee (IEC) was taken prior to the initiation of the study (approval letter no. EC/OA-28/2018). A total number of 100 cases of more than 20 years of age brought for postmortem examination were studied from February 2019 to February 2020. The cases of more than 20 years old brought for medicolegal postmortem examination whose age was confirmed by documentary evidence like birth certificates or Government approved identification cards were included in the study. Cases having a deformed or fractured skull that may hinder the study of suture closure or cranium revealing Wormian bones were omitted. Also not included were unidentified, unclaimed individuals where specific age, sex, and community cannot be determined.

The cranium was thoroughly examined and dissected by standard forensic autopsy procedures. After reflection of the scalp from the skull, the temporal muscles were dissected, and the periosteum was denuded. Then, the sutures, namely, coronal, sagittal, and lambdoid were examined ectocranially. The skull was opened with an oscillating saw. The frontal sawing point was two finger breadths above the supraorbital ridge. On both sides, the temporal point was taken at the top of the ear. The posterior occipital point was two finger breadths above the external protuberance. After sawing the skull, the chisel was rotated in the frontal saw line. The skull cap was carefully removed from the dura. After opening the skull with an oscillating saw, the coronal and sagittal sutures were examined endocranially on the inner surface of the skull cap. The lambdoid suture was studied in situ, endocranially. The degree of obliteration of sutures was scored ectocranially as well as endocranially, as done by Acsardi and Nemeskeri (Table 1) [5].

**Table 1:** Acsadi and Nemeskeri complex method

Suture grade	Suture status
0	There is still little space left between edges of adjoining bones.
1	Incipient closure. Clearly visible as a continuous often zigzagging line.
2	Closure in process. Line thinner, fewer zigzags, interrupted by complete closure
3	Advanced closure. Only pits indicate where the suture is located
4	Closed. Even location cannot be recognized

Division of sutures was done in 16 parts both ectocranially and endocranially. The coronal suture was divided into three parts on the right side (CR1, CR2, CR3) and three parts on the left side (CL1, CL2, CL3), sagittal suture in four parts (S1, S2, S3, S4) and lambdoid suture again in three parts on the right (LR1, LR2, LR3) and on the left side (LL1, LL2, LL3) [6].

Relevant data obtained were incorporated on a prescribed proforma. Data were then entered into a Microsoft Excel sheet. IBM SPSS Statistics for Windows, Version 21 (Released 2012; IBM Corp., Armonk, New York, United States) was used to analyze the data. Descriptive statistics were used for continuous data in terms of mean and standard deviation, and categorical data were presented by frequency and percentages. An independent t-test was done to find out the mean difference between the right and left sides of suture closure in total subjects for the ectocranial and endocranial surfaces. An independent t-test was also performed to find the difference in the mean change in ectocranial and endocranial sutural obliteration. The Spearman rank correlation test was carried out to find out the relationship between the age and score of suture closure both ectocranially and endocranially in total subjects. A probability value of less than 0.05 was considered statistically significant in each of the abovementioned methods of statistical evaluation.

## Results

In the present study, the age of the subjects varied from 20 to 95 years with a mean age of 35.98 ± 16.65 years. Age-wise distribution of cases is depicted in Table 2. Males constituted a majority and comprised 71% compared to females who were only 29 %.

**Table 2:** Age distribution

Age groups (years)	Frequency (N)	Percent (%)
20-29	45	45.0
30-39	22	22.0
40-49	13	13.0
50-59	8	08.0
>60	12	12.0
Total	100	100

Only four subjects showed complete obliteration of coronal suture ectocranially, which was in stage 4 of obliteration (two subjects were in the age group of 40-50 years and two in the age group of above 60 years). The mean score of ectocranial coronal suture obliteration in different age groups is depicted in Table 3. On comparing the mean ectocranial scores of 100 subjects by applying an independent t-test, no significant difference was observed in the obliteration of coronal sutures on the right and left sides (p-value 0.51).

**Table 3:** Mean score of ectocranial coronal suture obliteration in different age groups ECCR1: Ectocranial coronal suture right side part 1; ECCR2: ectocranial coronal suture right side part 2; ECCR3: ectocranial coronal suture right side part 3; ECCL1: ectocranial coronal suture left side part 1; ECCL2: ectocranial coronal suture left side part 2; ECCL3: ectocranial coronal suture left side part 3

Age group (years)	Parameter	ECCR1	ECCR2	ECCR3	ECCL1	ECCL2	ECCL3
20-29	N	45	45	45	45	45	45
	Mean	1.11	1.07	1.11	1.11	1.09	1.11
	S.D	0.885	0.809	0.647	0.885	0.821	0.647
30-39	N	22	22	22	22	22	22
	Mean	1.64	1.50	1.41	1.59	1.68	1.45
	S.D	0.658	0.673	0.590	0.666	0.780	0.510
40-49	N	13	13	13	13	13	13
	Mean	2.08	1.69	1.54	1.85	1.85	1.38
	S.D	0.641	0.480	0.877	0.376	0.801	0.506
50-59	N	8	8	8	8	8	8
	Mean	1.75	1.38	1.38	1.75	1.38	1.25
	S.D	0.463	0.518	0.518	0.463	0.518	0.463
60+	N	12	12	12	12	12	12
	Mean	2.08	1.92	1.50	2.08	2.08	1.58
	S.D	0.669	0.900	0.905	0.289	0.996	0.900
Total	N	100	100	100	100	100	100
	Mean	1.52	1.37	1.30	1.48	1.46	1.29
	S.D	0.847	0.787	0.704	0.785	0.881	0.640

Endocranially, 16 out of 100 subjects showed complete obliteration of coronal sutures. Out of 16 subjects, six subjects were above 60 years old; four subjects were in the 50-60-year age group; and six subjects were in the 40-50-year age group. The mean score of endocranial coronal suture obliteration in different age groups is depicted in Table 4. No statistically significant difference was observed in the obliteration of right and left side coronal sutures endocranially (p-value 0.528).

**Table 4:** Mean score of endocranial coronal suture obliteration in different age groups ENCR1: Endocranial coronal suture right side part 1; ENCR2: endocranial coronal suture right side part 2; ENCR3: endocranial coronal suture right side part 3; ENCL1: endocranial coronal suture left side part 1; ENCL2: endocranial coronal suture left side part 2; ENCL3: endocranial coronal suture left side part 3

Age group (years)	Parameter	ENCR1	ENCR2	ENCR3	ENCL1	ENCL2	ENCL3
20-29	N	45	45	45	45	45	45
	Mean	2.42	2.44	2.40	2.42	2.44	2.44
	S.D	0.621	0.659	0.618	0.621	0.659	0.659
30-39	N	22	22	22	22	22	22
	Mean	2.68	2.59	2.59	2.68	2.64	2.55
	S.D	0.646	0.590	0.590	0.646	0.581	0.510
40-49	N	13	13	13	13	13	13
	Mean	2.77	2.77	2.77	2.77	2.77	2.77
	S.D	0.725	0.725	0.725	0.725	0.725	0.725
50-59	N	8	8	8	8	8	8
	Mean	3.00	3.00	3.00	3.00	3.00	3.00
	S.D	0.756	0.756	0.756	0.756	0.756	0.756
60+	N	12	12	12	12	12	12
	Mean	3.50	3.50	3.50	3.50	3. 50	3.50
	S.D	0.522	0.522	0.522	0.522	0.522	0.522
Total	N	100	100	100	100	100	100
	Mean	2.70	2.69	2.67	2.70	2.70	2.68
	S.D	0.718	0.720	0.711	0.718	0.718	0.709

On observing the pattern of ectocranial sagittal suture obliteration in all subjects, it was noticed that part 3 of sagittal suture (S3) appears to obliterate slower (mean score 1.39) in comparison to S1 (mean score 1.68), S2 (mean score 1.56), and S4 (mean score 1.44) in all age groups (Table 5).

**Table 5:** Mean score of ectocranial sagittal suture obliteration in different age groups ECS1 = Ectocranial sagittal suture part 1; ECS2: ectocranial sagittal suture part 2; ECS3: ectocranial sagittal suture part 3; ECS4: ectocranial sagittal suture part 4

Age group (years)	Parameter	ECS1	ECS2	ECS3	ECS4
20-29	N	45	45	45	45
	Mean	1.44	1.27	1.13	1.16
	S.D	0.755	0.539	0.457	0.475
30-39	N	22	22	22	22
	Mean	1.73	1.45	1.23	1.36
	S.D	0.456	0.510	0.528	0.581
40-49	N	13	13	13	13
	Mean	1.77	1.77	1.46	1.62
	S.D	0.439	0.927	0.660	0.961
50-59	N	8	8	8	8
	Mean	1.75	1.63	1.63	1.63
	S.D	0.463	0.518	0.744	0.744
60+	N	12	12	12	12
	Mean	2.33	2.58	2.42	2.33
	S.D	0.888	1.084	.379	1.303
Total	N	100	100	100	100
	Mean	1.68	1.56	1.39	1.44
	S.D	0.709	0.783	0.790	0.808

The endocranial sagittal suture obliterates earlier in S1 and S2 subparts followed by S4 and S3 (mean score of 2.76, 2.76, 2.71, and 2.68 respectively). Near complete obliteration of the endocranial sagittal suture (mean score 3.50) was observed in the age group of above 60 years (Table 6).

**Table 6:** Mean score of endocranial sagittal suture obliteration in different age groups ENS1: Endocranial sagittal suture part 1; ENS2: endocranial sagittal suture part 2; ENS3: endocranial sagittal suture part 3; ENS4: endocranial sagittal suture part 4

Age group (years)	Parameter	ENS1	ENS2	ENS3	ENS4
20-29	N	45	45	45	45
	Mean	2.51	2.51	2.40	2.44
	S.D	0.661	0.661	0.580	0.586
30-39	N	22	22	22	22
	Mean	2.59	2.64	2.50	2.59
	S.D	0.503	0.581	0.512	0.590
40-49	N	13	13	13	13
	Mean	2.92	2.92	2.92	2.85
	S.D	0.641	0.641	0.641	0.555
50-59	N	8	8	8	8
	Mean	3.25	3.13	3.13	3.13
	S.D	0.463	0.354	0.354	0.354
60+	N	12	12	12	12
	Mean	3.50	3.50	3.50	3.50
	S.D	0.522	0.522	0.522	0.522
Total	N	100	100	100	100
	Mean	2.76	2.76	2.68	2.71
	S.D	0.659	0.659	0.646	0.632

In all of the 100 subjects, ectocranially, lambdoid sutures showed that the L1 subpart (LR1 mean 1.45 and LL1 mean 1.47) appeared to obliterate early (Table 7). The maximum mean score of 2.00 was observed in ectocranial obliteration of lambdoid sutures in the age group of 60 years and above. On comparing the mean ectocranial scores of 100 subjects by applying an independent t-test, no significant difference was observed in the obliteration of lambdoid sutures on the right and left sides (p-value 0.739).

**Table 7:** Mean score of ectocranial lambdoid suture obliteration in different age groups ECLR1: Ectocranial lambdoid suture right side part 1; ECLR2: ectocranial lambdoid suture right side part 2; ECLR3: ectocranial lambdoid suture right side part 3; ECLL1: ectocranial lambdoid suture left side part 1; ECLL2: ectocranial lambdoid suture left side part 2; ECLL3: ectocranial lambdoid suture left side part 3

Age Group	Parameter	ECLR1	ECLR2	ECLR3	ECLL1	ECLL2	ECLL3
20-29	N	45	45	45	45	45	45
	Mean	1.31	1.20	1.18	1.31	1.20	1.18
	S.D	0.468	0.457	0.442	0.468	0.457	0.442
30-39	N	22	22	22	22	22	22
	Mean	1.41	1.36	1.36	1.41	1.32	1.32
	S.D	0.590	0.581	0.581	0.590	0.568	0.568
40-49	N	13	13	13	13	13	13
	Mean	1.54	1.46	1.46	1.62	1.38	1.46
	S.D	0.519	0.519	0.519	0.506	0.506	0.519
50-59	N	8	8	8	8	8	8
	Mean	1.38	1.50	1.50	1.50	1.50	1.63
	S.D	0.518	0.535	0.535	0.535	0.535	0.518
60+	N	12	12	12	12	12	12
	Mean	2.00	2.00	1.83	2.00	2.00	1.92
	S.D	1.206	1.206	1.267	1.206	1.206	1.240
Total	N	100	100	100	100	100	100
	Mean	1.45	1.39	1.36	1.47	1.37	1.37
	S.D	0.657	0.665	0.659	0.658	0.661	0.661

The endocranial lambdoid suture showed no major difference between left and right-side subparts obliteration. LR 1 and LL1 obliterate earlier (mean 2.69 each) followed by LR2, LR3, LL2, and LL3. Near complete obliteration of the endocranial lambdoid suture (mean score 3.50) was observed in the age groups of above 60 years (Table 8). No statistically significant difference was observed in the obliteration of lambdoid sutures on the right and left sides endocranially.

**Table 8:** Mean score of endocranial lambdoid suture obliteration in different age groups ENLR1: Endocranial lambdoid suture right side part 1; ENLR2: endocranial lambdoid suture right side part 2; ENLR3: endocranial lambdoid suture right side part 3; ENLL1: endocranial lambdoid suture left side part 1; ENLL2: endocranial lambdoid suture left side part 2; ENLL3: endocranial lambdoid suture left side part 3

Age Group	Parameter	ENLR1	ENLR2	ENLR3	ENLL1	ENLL2	ENLL3
20-29	N	45	45	45	45	45	45
	Mean	2.51	2.49	2.49	2.51	2.49	2.49
	S.D	0.626	0.589	0.589	0.626	0.589	0.589
30-39	N	22	22	22	22	22	22
	Mean	2.50	2.50	2.50	2.50	2.50	2.50
	S.D	0.512	0.512	0.512	0.512	0.512	0.512
40-49	N	13	13	13	13	13	13
	Mean	2.77	2.77	2.77	2.77	2.77	2.77
	S.D	0.599	0.599	0.599	0.599	0.599	0.599
50-59	N	8	8	8	8	8	8
	Mean	2.88	2.88	2.88	2.88	2.88	2.88
	S.D	0.641	0.641	0.641	0.641	0.641	0.641
60+	N	12	12	12	12	12	12
	Mean	3.50	3.50	3.50	3.50	3.50	3.50
	S.D	0.522	0.522	0.522	0.522	0.522	0.522
Total	N	100	100	100	100	100	100
	Mean	2.69	2.68	2.68	2.69	2.68	2.68
	S.D	0.662	0.649	0.649	0.662	0.649	0.649

When we compared three sutures ectocranially, the overall sagittal suture obliterates early followed by the coronal suture and then the lambdoid suture (mean 1.517, 1.403, and 1.401 respectively). Endocranially, the overall sagittal suture obliterates early followed by the coronal suture and then the lambdoid suture (mean 2.727, 2.690, and 2.683) (Table 9).

**Table 9:** Mean score of ectocranial and endocranial obliteration of three sutures

Age	Parameter	CORONAL SUTURE		SAGITTAL SUTURE		LAMBDOID SUTURE	
		ECTOCRANIAL	ENDOCRANIAL	ECTOCRANIAL	ENDOCRANIAL	ECTOCRANIAL	ENDOCRANIAL
20-29	N	45	45	45	45	45	45
	Mean	1.100	2.429	1.250	2.466	1.229	2.496
	S.D	0.700	0.618	0.442	0.578	0.399	0.597
30-39	N	22	22	22	22	22	22
	Mean	1.545	2.621	1.443	2.579	1.363	2.500
	S.D	0.522	0.527	0.326	0.484	0.531	0.511
40-49	N	13	13	13	13	13	13
	Mean	1.730	2.769	1.653	2.903	1.487	2.769
	S.D	0.448	0.725	0.657	0.608	0.448	0.599
50-59	N	8	8	8	8	8	8
	Mean	1.479	3.000	1.656	3.156	1.500	2.875
	S.D	0.165	0.755	0.498	0.351	0.427	0.640
60+	N	12	12	12	12	12	12
	Mean	1.875	3.500	2.416	3.500	1.958	3.500
	S.D	0.599	0.522	1.088	0.522	1.210	0.522
Total	N	100	100	100	100	100	100
	Mean	1.403	2.690	1.517	2.727	1.401	2.683
	S.D	45	0.695	45	0.639	45	0.651

On comparing the mean ectocranial and mean endocranial scores of 100 subjects by applying an independent t-test, a highly significant difference was observed in the obliteration of all three sutures (Table 10).

**Table 10:** Statistical analysis of obliteration of ectocranial and endocranial sutures

Suture	n	t	Df	Mean difference	Std error difference	95% Confidence interval of difference		Sig. (two-tailed)
						Lower	Upper	
Coronal	100	-13.465	198	-1.28666	0. 09555	-1.47510	-1.09822	0.000
Sagittal	100	-13.074	198	-1.21000	0.09255	-1.39251	-1.02749	0.000
Lambdoid	100	-14.250	198	1.28166	0. 08994	-1.45903	-1.10429	0.000

On correlating ectocranial sutures and endocranial sutures and age at death, a highly significant correlation was found in all the subjects combined (p-value 0.000). However, no significant correlation (p-value >0.05) was found in ectocranial and endocranial sagittal sutures in individual age groups (Table 11).

**Table 11:** Spearman rank correlation coefficient and level of significance between the mean score of the obliteration of ectocranial and endocranial sutures in various age groups **Correlation is significant at the 0.01 level (two-tailed)

AGE GROUP	N	Test	ECTOS/ ENDOS	ECTOCR/ ENDOCR	ECTOCL/ ENDOCL	ECTOLR/ ENDOLR	ECTOLL/ ENDOLL
20-29	45	Corr. Coeff	0.213	0.989**	0.984**	0.977**	0.984**
		Sig. 2 tailed	0.159	0.000	0.000	0.000	0.000
30-39	22	Corr. Coeff	0.300	0.970**	0.968**	0.968**	0.968**
		Sig. 2 tailed	0.175	0.000	0.000	0.000	0.000
40-49	13	Corr. Coeff	0.220	0.960**	0.960**	0.908**	0.960**
		Sig. 2 tailed	0.470	0.000	0.000	0.000	0.000
50-59	08	Corr. Coeff	0.274	0.900**	0.900**	0.900**	0.900**
		Sig. 2 tailed	0.511	0.002	0.002	0.002	0.002
>60	12	Corr. Coeff	0.415	0.984**	0.833**	0.955**	0.941**
		Sig. 2 tailed	0.180	0.000	0.001	0.000	0.000
Total	100	Corr. Coeff	0.373**	0.980**	0.956**	0.951**	0.966**
		Sig. 2 tailed	0.000	0.000	0.000	0.000	0.000

## Discussion

Age determination is an integral part of the medicolegal autopsy, particularly in cases of unidentified dead bodies. It aids in the identification of an individual in the event of mass disasters that are not unforeseen these days. Estimation of the age of a living individual is also asked routinely by law enforcement agencies in civil as well as criminal cases when no valid documentary proof is available with the individual. Even when the birth certificate and school records provide the age of a person, still its scientific confirmation is required by a court of law and specific administrative departments. Estimation of age up to 25 years does not pose any challenge for forensic experts, as age can be determined within the range of one to two years with a certain degree of certainty up to 25 years of age by general physical, dental, and radiological findings of ossification of long bones. Even in dead, epiphyseal-diaphyseal fusion can be seen and age can be determined without any difficulty up to 25 years of age. The challenge for forensic experts is the determination of age after 25 years both in dead and living. Cranial sutures are one of the criteria which are used for the determination of age. The pattern of obliteration of cranial sutures helps in corroborating the age of an individual in the later years of life.

In the present study, ectocranially, only four subjects showed complete obliteration. Endocranially, 16 subjects showed complete obliteration of coronal sutures. It signifies that lapsed union was more evident ectocranially than endocranially. Consistent with our findings, Jangjetriew et al. observed that the age at which endocranial sutures begin to obliterate precedes the age at which ectocranial sutures close [7]. Krogman also documented that due to the phenomenon of 'Lapsed union' ectocranially, the state of endocranial obliteration must take precedence in any evaluation of suture age [6]. Todd and Lyon also observed that sutures obliterates earlier endocranially, rather than ectocranially [8]. Ectocranial obliteration progresses more slowly and shows more individual variation than endocranial obliteration. Ectocranial suture obliteration was never as complete as endocranial obliteration [8]. Masih et al. also observed in their study that the commencement of obliteration of the endocranial coronal suture started at 25-30 years, and its completion in either sex was completed at the age of 46-50 years. They also concluded that obliteration of the endocranial suture was more reliable than the ectocranial suture [9].

On observing the pattern of sagittal suture obliteration in all subjects in the current study, near-complete obliteration (mean score 3.50) was observed in the age of above 60 years, endocranially. Ectocranially, only five subjects showed complete obliteration of the sagittal suture, while, endocranially, 14 subjects showed complete obliteration. Observations made by Hershkovitz et al. were somewhat similar to our findings, as they concluded that the sagittal suture could not be used for aging the skeleton [10]. Endocranial suture obliteration begins before ectocranial suture obliteration does, and Jangjetriew et al. discovered no significant variation in this regard between the sexes [7]. Masih et al. concluded that endocranial sagittal suture obliteration was completed at the age of 46-50 years in both males and females [9]. While on ectocranium, completion of obliteration took place at the age of 51-55 years. The obliteration occurred 5-10 years earlier on endocranial as compared to ectocranium on all vault sutures. They concluded that obliteration of the endocranial suture was more reliable than the ectocranial suture [9].

Near-complete endocranial obliteration of the lambdoid suture (mean 3.50) was observed in ages above 60 years. In the present study, complete obliteration of the lambdoid suture was observed in two cases only, ectocranially. Endocranially, 10 subjects showed complete obliteration of the lambdoid suture. Kumar et al. also observed in their study that ectocranially and endocranially, no significant difference between the right and left sides [11]. Their study results were corroborative of our findings.

The pattern of obliteration observed in various studies conducted in different regions is depicted in Table 12.

**Table 12:** Pattern of obliteration observed in comparison to various studies

Sr. No.	Author	Year	Place of study	No. of cases	The pattern of obliteration in order of fusion		
1	Parsons & Box [12]	1905	United Kingdom	82	Coronal	Sagittal	Lambdoid
2	Todd and Lyon [13]	1924	American white males	514	Sagittal (endocranial)	Coronal (endocranial)	Lambdoid (endocranial)
3	Ramanan et al.[14]	2016	Tamil Nadu, India	100	Coronal (endocranial)	Sagittal (endocranial)	Lambdoid (endocranial)
4	Tiwari et al. [15]	2018	Madhya Pradesh, India	500	Sagittal (ectocranial and endocranial)	Coronal (ectocranial &endocranial)	Lambdoid (ectocranial &endocranial)
5	Present study	2019	Haryana, India	100	Sagittal (ectocranial)	Coronal (ectocranial)	Lambdoid (ectocranial)
					Sagittal (endocranial)	Coronal (endocranial)	Lambdoid (endocranial)

On correlating ectocranial sutures and endocranial sutures and age at death in all the cases through sagittal, right and left coronal, and lambdoid by applying the Spearman rank correlation coefficient, a highly significant correlation was found in all the subjects combined (p-value 0.000). However, no significant correlation (p-value >0.05) was found in ectocranial and endocranial sagittal sutures in individual age groups. Tiwari et al. found a statistically significant correlation between ectocranial and endocranial sagittal suture in the 20-29, 30-39, 40-49, and >70 age groups, but not in the 50-59 and 60-69 age groups [15]. However, they conducted their study on 500 cadavers in comparison to 100 cadavers in the present study, which might be the reason for the different results. Similarly, Perizonius et al. conducted a survey of 256 human crania from the Amsterdam collection in the Netherlands and concluded that the mean endocranial closure stage is correlated (P < 0.001) with age in the ages below 50 years. They observed that all sutures especially the coronal exhibited a significant degree of positive correlation with age, in the 20-49 years of age [16]. Ramanan et al. in their research also observed that there was some correlation between endocranial suture obliteration and age up to 50 years' age group, after that, there was no significant correlation [14]. Sahni et al. reached the conclusion that the obliteration of the various segments of the three primary sutures of the cranium is so random that it neither aids in estimating the age of the deceased nor provides evidence for determining the age of skeletal remains [17]. Krogman had explicitly documented that the estimation of the age of the skull via suture obliteration was not reliable. If the head is the only part present, then age estimation can be done best by this. If other bones are present, then suture age may become at best, partially corroborative [6].

Limitations of the study

One of the few limitations of the study was its small sample size which is 100 cases. Also, the number of cases in each age group was also dissimilar. The number should be equal in each age group to arrive at an affirmative conclusion. Migration, relocation, and interracial marriage have all contributed to genetic mixing across racial lines. The current research did not take these details into account.

## Conclusions

Ectocranially and endocranailly, the overall sagittal suture obliterates earlier followed by the coronal suture and then the lambdoid suture. Near-complete obliteration with a mean score of 3.50 was observed in all three sutures endocranially, in the age group of above 60 years. Keeping in view the results of the present study regarding cranial vault suture obliteration, we concluded that obliteration on the endocranial surface is more reliable than on the ectocranial surface. No statistically significant difference exists on the obliteration of sutures on the right and left sides of coronal and lambdoid sutures. The lapsed union was evident in all three sutures ectocranially. Both ectocranially and endocranially, suture obliteration has some correlation with age. Endocranial suture obliteration can be used as a corroborative tool for age estimation. Further studies should be contemplated with a larger sample size and equal distribution of cases in all the age groups for an affirmative analysis and implementation of obliteration of cranial sutures for estimation of age.
